# Medical News

**Published:** 1855-02

**Authors:** 


### MEDICAL NEWS.

Philadelphia County Medical Society.—At a Meeting, held January 17th, 1855, the following Officers and Delegates were elected for the present year, viz:

*President*—D. Francis Condie.

*Vice Presidents*—Wilson Jewell and Francis West.

*Recording Secretary*—Robert P. Thomas.

*Assistant Secretary*—J. Aitken Meigs.

*Corresponding Secretary*—Isaac Remington.

*Treasurer.*—Wm. Byrd Page.

*Censors.*—John B. Biddle, Anthony E. Stocker, Lewis Rodman, Wm. N. Johnson and Samuel Lewis.

*Delegates to the American Medical Association*—John Bell, Thomas F. Betton, D. Francis Condie, John Conrey, Samuel L. Hollingsworth, Samuel Jackson (Spruce street), Benjamin S. Janney, Wilson Jewell, William H. Klapp, D. Paul Lajus, John F. Lamb, R. La Roche, Samuel Lewis, Arnold Naudain, Wm. Byrd Page, Lewis Rodman, Anthony E. Stocker, Robert P. Thomas, Ellwood Wilson, Caspar Wister, Thomas H. Yardley and Jacob S. Zorns.

*Delegates to the State Medical Society*—T. Hewson Bache, John B. Biddle, Thomas F. Betton, Joseph Carson, Benjamin H. Coates, D. Francis Condie, John Conrey, G. Emerson, James V. Emlen, David Gilbert, Robert A. Given, Edward Hartshorne, Isaac Hays, Adinell Hewson, Samuel L. Hollingsworth, Nathan L. Hatfield, Alexander C. Hart, Abram Helffenstein, John H. Ingham, Samuel Jackson (Spruce street), Benjamin S. Janney, Wilson Jewell, Alfred L. Kennedy, Wm. H. Klapp, R. La Roche, Samuel Lewis, John F. Lamb, John H. B. McClellan, John K. Mitchell, S. Weir Mitchell, William Mayburry, John Neill, Arnold Naudain, Joseph Pancoast, John M. Pugh, Alfred Stillé, Francis G. Smith, Robert P. Thomas, Francis West, Ellwood Wilson, Caspar Wister, George B. Wood, Thos. H. Yardley and Jacob S. Zorns. From the Minutes,

Robert B. Thomas, Recording Secretary.

At the Annual Meeting of the Northern Medical Association, held Jan. 12, 1855, the following Officers were elected for the ensuing year.

*President*—Dr. M. B. Smith.

*Vice President*—Dr. John Rhein.

*Treasurer*—Dr. J. Henry Smaltz.

*Recording Secretary*—Dr. Levi Curtis.

*Reporting Secretaries*—Drs. Joseph R. Bryan and Charles Wittig.

*Corresponding Secretary*—Dr. William Mayburry.

*Counsellors*—Drs. R. H. Townsend, N. L. Hatfield, R. J. Levis, George J. Zeigler and John Uhler.

*Delegates to the American Medical Association*—Drs. N. L. Hatfield, R. H. Townsend, J. Henry Smaltz, John Rhein and L. Curtis.

*Selective Committee*—Drs. George J. Zeigler, R. J. Levis, J. Henry Smaltz, Joseph R. Bryan and L. Curtis.

LEVI CURTIS, Recording Secretary.

---

ERRATUM.—In Dr. H. H. Smith's article on "Gastrotomy," in our last Number, the word *lump-like*, on page 4, should read *lymph-like.*

---

*Abstract of Meteorological Observations for December*, 1854, *made at Philadelphia, Pa. Latitude* 39° 57′ 28″ *N., Longitude* 75° 10′ 40″ *W. from Greenwich.* By PROF. JAMES A. KIRKPATRICK.

| 1854. Dec. | Barometer. Daily Mean | Barometer. Mean Daily Range. | Thermom. Daily Mean | Thermom. Mean Daily Range | Dew Point 2 P.M. | Rel. Humid. 2 P. M. | Rain. and melted Snow. | Prevailing Winds. | Remarks. |
|---|---|---|---|---|---|---|---|---|---|
| | Inches. | Inches. | Deg. | Deg. | Deg. | Hunds. | Inch. | Points. | |
| 1 | 29.885 | .104 | 31.7 | 7.0 | 26.0 | 0.71 | | (Var.) | M. clear; aft. and ev. cloudy. |
| 2 | 29.910 | .083 | 34.5 | 4.2 | 25.8 | .53 | | (Var.) | M. cloudy; aft. and ev. clear. |
| 3 | 29.426 | .484 | 35.7 | 2.5 | 41.0 | 1.00 | 0.620 | N. | Cloudy. *Rain* all day, ev. *Snow. Bar. lowest* 29.244. |
| 4 | 29.344 | .247 | 27.5 | 8.2 | 20.0 | .66 | | NW. | M. and aft. cloudy; ev. clear. |
| 5 | 29.521 | .143 | 24.3 | 3.2 | 20.0 | .66 | | W.NW. | Cloudy. |
| 6 | 29.569 | .104 | 30.0 | 5.7 | 30.7 | .88 | | SW. | Cloudy. |
| 7 | 29.514 | .134 | 30.3 | 7.0 | 23.3 | .52 | | W. | M. and aft. cloudy; ev. clear. |
| 8 | 30.025 | .511 | 22.0 | 8.7 | 15.7 | .50 | | NW. | Clear. |
| 9 | 30.380 | .355 | 25.7 | 5.0 | 23.0 | .69 | | SW. | Clear. |
| 10 | 30.165 | .215 | 36.7 | 11.0 | 32.7 | .66 | | SW. | M. clear; aft. and ev. cloudy. Rain during the night. |
| 11 | 29.963 | .202 | 36.3 | 6.3 | 30.7 | .65 | 0.696 | NW. | M. and aft. cloudy; ev. clear. |
| 12 | 30.027 | .064 | 31.5 | 4.8 | 28.3 | .79 | | NW. | M. and aft. cloudy; ev. clear. |
| 13 | 29.921 | .111 | 36.0 | 5.8 | 30.3 | .73 | | SW. | M. & aft. cl'y; ev. cl'r. *Th. hig'st* 48°. |
| 14 | 29.855 | .065 | 40.0 | 4.7 | 38.7 | .70 | | SW. | M. and aft. cloudy; ev. clear. |
| 15 | 29.719 | .137 | 39.0 | 4.3 | 36.0 | .75 | | NE. | M. clear; aft. and ev. cloudy. |
| 16 | 29.650 | .191 | 41.0 | 2.0 | 35.7 | .68 | | NW. | Cloudy. |
| 17 | 29.849 | .198 | 32.3 | 8.7 | 25.0 | .70 | | NE. | Cloudy. |
| 18 | 29.848 | .058 | 27.3 | 2.3 | 23.3 | .77 | 0.020 | N.NW. | M. *Snow*, aft. cloudy; ev. clear. |
| 19 | 29.946 | .098 | 17.3 | 10.0 | 14.0 | .59 | | N. | Clear. |
| 20 | 29.935 | .015 | 14.3 | 4.3 | 12.0 | .57 | | W. | Clear. *Therm. lowest* 6°. |
| 21 | 29.871 | .064 | 29.3 | 15.0 | 28.3 | .79 | 0.052 | (Var.) | M. *Snow*: cloudy. |
| 22 | 30.174 | .303 | 30.8 | 6.5 | 27.7 | .62 | | (Var.) | Cloudy. |
| 23 | 30.477 | .304 | 20.5 | 10.7 | 20.0 | .80 | 0.149 | NE. | Cl'y; ev. rain. *Bar. highest* 30.504. |
| 24 | 30.154 | .323 | 34.3 | 13.8 | 33.7 | .89 | 0.178 | SW. | M. rain, cloudy; ev. fog. |
| 25 | 30.034 | .120 | 35.3 | 1.7 | 36.7 | .90 | | SW. | M. and aft. cloudy; ev. clear. |
| 26 | 29.939 | .094 | 38.5 | 3.2 | 40.0 | .88 | 1.354 | NE. | Cloudy; m. fog; aft. & night rain. |
| 27 | 29.794 | .146 | 44.3 | 5.8 | 44.7 | .92 | | N. | Cloudy. |
| 28 | 29.794 | .067 | 40.2 | 4.2 | 37.7 | .90 | 0.116 | NE. | Cloudy; ev. fog; aft. and night rain |
| 29 | 29.750 | .131 | 31.3 | 8.8 | 24.0 | .69 | 0.116 | NW. | M. *Rain* till 8, *Snow* till 12. |
| 30 | 29.936 | .190 | 22.0 | 9.3 | 15.7 | .50 | | NW. | Clear. |
| 31 | 30.063 | .127 | 29.3 | 7.3 | 26.0 | .71 | | NW. | Clear. |
| Means for Dec. 1854 | 29.885 | .174 | 31.3 | 6.5 | 28.0 | .74 | 3.185 | N. 51° W., 63–100. | |
| 3yrs | 29.911 | | 35.2 | | 31.7 | | 3.510 | N. 47½° W. 51–100. | |

Monthly Range of Barometer 1.260, and of the Thermometer 42°.

*Abstract of Meteorological Observations made at Philadelphia, Pa., Latitude* 39° 57′ 28″ *N., Longitude* 75° 10′ 40″ *from Greenwich, for the year* 1854. By PROF. JAMES A. KIRKPATRICK.

| 1854. | Barometer, reduced to 32° F. | | | | Thermometer. | | | | Dew Point, 2 P.M. | | | | Rain & Melted Snow. | Winds. Monthly Resultant. | Remarks. |
|---|---|---|---|---|---|---|---|---|---|---|---|---|---|---|---|
| | Mean Inch. | Highest Inches. | Lowest Inches. | Range Inches. | M. Deg. | H. Deg. | L. Deg. | R. Deg. | M. Deg. | H. Deg. | L. Deg. | R. Deg. | Inches. | No. of times in 1000. | |
| January | 30.008 | 30.471 | 29.384 | 1.087 | 30.42 | 58 | 11½ | 46½ | 31.82 | 58.0 | 21.0 | 37.0 | 2.320 | N. 88° W. .559 | The warmest day was July |
| February | 29.957 | 30.404 | 29.432 | .972 | 33.42 | 61 | 16 | 45 | 33.04 | 55.3 | 24.7 | 30.6 | 4.200 | N. 71° W. .461 | 21st, mean temperature, |
| March | 29.842 | 30.260 | 29.158 | 1.102 | 42.22 | 75 | 22 | 53 | 34.63 | 57.7 | 17.0 | 40.7 | 1.625 | N. 53° W. .543 | 91.3°. |
| April | 29.872 | 30.518 | 29.249 | 1.269 | 50.08 | 85 | 28 | 57 | 39.29 | 52.0 | 23.7 | 28.3 | 8.145 | West .378 | Thermometer highest July |
| May | 29.847 | 30.153 | 29.585 | .568 | 64.25 | 84 | 35 | 49 | 49.09 | 69.0 | 26.7 | 42.3 | 7.299 | S. 85° W. .140 | 21st, 100½°, 3½ P. M. |
| June | 29.824 | 30.098 | 29.519 | .549 | 71.63 | 96 | 49 | 47 | 54.82 | 72.7 | 37.7 | 35.0 | 3.441 | S. 76° W. .517 | Coldest day, December 20, |
| July | 29.926 | 30.118 | 29 711 | .407 | 79.11 | 100½ | 63 | 37½ | 59.22 | 72.0 | 47.7 | 24.3 | 3.837 | N. 71° W. .410 | mean temperature 14.3°. |
| August | 29.946 | 30.185 | 29.701 | .484 | 75.49 | 95 | 56 | 39 | 56.70 | 71 0 | 45.7 | 25.3 | 0.918 | N. 81° W. .212 | Thermometer lowest Dec. |
| September | 30.020 | 30.388 | 29.657 | .731 | 70.02 | 94 | 47 | 47 | 56.54 | 69.7 | 36.0 | 33.7 | 4.883 | West .178 | 20th, 6°. |
| October | 29.994 | 30.330 | 29.431 | .891 | 59.15 | 79 | 34 | 45 | 47.19 | 67.7 | 29.3 | 38.4 | 1 918 | N. 70° W. .320 | Barometer highest, 30.518, |
| November | 29.831 | 30.369 | 29.257 | 1.112 | 46 13 | 69 | 27 | 42 | 37.41 | 62.7 | 24.3 | 38.4 | 3.460 | S. 85° W. .614 | on the 3d of April. |
| December | 29.885 | 30.504 | 29.244 | 1.260 | 31.26 | 48 | 6 | 42 | 27 96 | 44.7 | 12 0 | 32.7 | 3.185 | N. 51° W. .629 | |
| Annual means | 29.913 | 30.518 | 29.158 | 1.360 | 51.43 | 100½ | 6 | 94½ | 43.98 | 72.7 | 12.0 | 60.7 | 45.23 | N. 77° W. .378 | Barometer lowest, 29.158, on the 17th of March. |
| Winter | 29.945 | 30.471 | 29.115 | 1.356 | 32.55 | 61 | 11 | 50 | 31.56 | 58.0 | 16.0 | 42.0 | 10.680 | N. 64° W. .496 | |
| Spring | 29.854 | 30.518 | 29.158 | 1.360 | 52.18 | 85 | 22 | 63 | 41.00 | 69.0 | 17.0 | 52.0 | 17.069 | N. 72° W. .356 | |
| Summer | 29.899 | 30.185 | 29.549 | .636 | 75.41 | 100½ | 49 | 51½ | 56.91 | 72.7 | 37.7 | 35.0 | 8.196 | S. 87° W. .268 | |
| Autumn | 29.948 | 30.388 | 29.257 | 1.131 | 58.43 | 94 | 27 | 67 | 47.05 | 69.7 | 24.3 | 45.4 | 10.261 | N. 82° W. .367 | |
| For 3 years | 29.920 | 30.709 | 28.895 | 1.804 | 54.04 | 100½ | -3 | 103½ | 43.47 | 75.0 | 12.0 | 63.0 | 44.80 | N. 74° W. .369 | |

